# The medieval bronze doors of San Zeno, Verona: combining material analyses and art history

**DOI:** 10.1186/s40494-024-01143-2

**Published:** 2024-01-25

**Authors:** M. Mödlinger, J. Bontadi, M. Fellin, M. Fera, M. Negri, J. Utz, G. Ghiara

**Affiliations:** 1 IMAREAL, Paris Lodron University Salzburg, Körnermarkt 13, 3500 Krems an der Donau, Austria; 2 CNR-IBE, Consiglio Nazionale delle Ricerche, Istituto per la Bioeconomia, via Francesco Biasi 75, 38098 San Michele all’Adige, Italy; 3 Institut Für Urgeschichte Und Historische Archäologie, Universität Wien, Franz-Klein-Gasse 1, 1190 Vienna, Austria; 4 DISAT, Politecnico di Torino, Corso Duca degli Abruzzi 24, 10129 Turin, Italy

**Keywords:** Chemical analyses, PCA, Portable ED-XRF, Wood anatomy, Mediaeval bronzes, Metal doors

## Abstract

The bronze doors of the Basilica of San Zeno in Verona, Italy, are a special case in art history research. They were made by several workshops during the twelfth century: stylistically, two to three workshops were assumed to produce the metal parts of the door. However, it is still unclear when exactly and if this interpretation can be supported by the chemical composition of the metal. In this research we aimed to verify the art history interpretation by identifying the alloy composition of each individual metal plate. The composition of the supporting wooden structures are discussed. A portable ED-XRF instrument and optical microscopes were used to analyse and document the doors non-invasively. The doors were also photographed to produce high resolution orthophotos and 3D models. We can confirm that the metal parts of the doors were made of leaded tin-bronze as well as leaded brass and mounted on a wooden structure mainly made of spruce and oak wood. Chemically, two/three different groups of alloys have been identified, which can be associated with two or three different workshops, and which largely correspond to the stylistic interpretation.

**Supplementary Information:**

The online version contains supplementary material available at 10.1186/s40494-024-01143-2.

## Introduction

Doors are generally made of wood. In special cases, they can be made of other materials or a combination of different materials. Since antiquity, some doors of temples or palaces were made of bronze, or a combination of bronze and wood, such as the doors of the *Cura Iulia* in Rome. This tradition was continued in particular by the Church in the Middle Ages. The surface of these metal doors was often richly decorated with scenes from the Old and New Testaments. While a small number of antique and early medieaval doors survive, the majority of these doors date from the eleventh and twelfth centuries [1]. Most of these doors—about 30 of them are known today—are found in Italy, followed by three in Germany and one each in Russia and Poland [1]. All of these doors are the study focus of an interdisciplinary Austrian research project. For the first time, material analyses on metal and wood are combined with a complete high-resolution photographic documentation of the doors and with historical and art historical research questions, providing information on materials used, technologies applied and the network of artists and donors involved.

The bronze doors from the eleventh and twelfth centuries were either made entirely of metal (such as the doors from Hildesheim or Canosa) or as a combination of wood and various copper alloys as is the case with the door of San Zeno, Verona. In both cases, the lost wax process was used to cast the doors or the individual metal parts that were later mounted on the wooden base. Since their production, some of the doors have been damaged by fire or earthquake, or their wooden base has been restored and replaced with a newer one [1]. As a result, the current order of the individual metal panels is not necessarily the original order. Another problem is the rearrangements of single plates after some of them had been stolen, or that the single plates had to be secured from recreational activities of kids.

In this study we focus on the bronze doors from San Zeno, Verona. They are a special case of doors that combine metal elements on a wooden support and are located on the main door of the basilica. They are 4.95 m high and about 3.82 m wide. The doors are placed on a wooden support on which 79 metal plates are applied (48 main square plates; 7 side decorative plates; 17 small square plates; 7 plates depicting different personalities), along with 88 decorative frame elements and 48 heads covering the joints of the latter. Some of the metal elements are perforated so that the wooden support is visible behind the panels. As the plates were (are) relatively easy to remove, the basilica had to notify only at the end of the 1970s the stealing of three heads (top left of plates C7, B8, C8) and a side element; others, as two small square plates, were lost during World War II (originally at the *Staatliche Museen,* Berlin) [1].

**Fig. 1:**
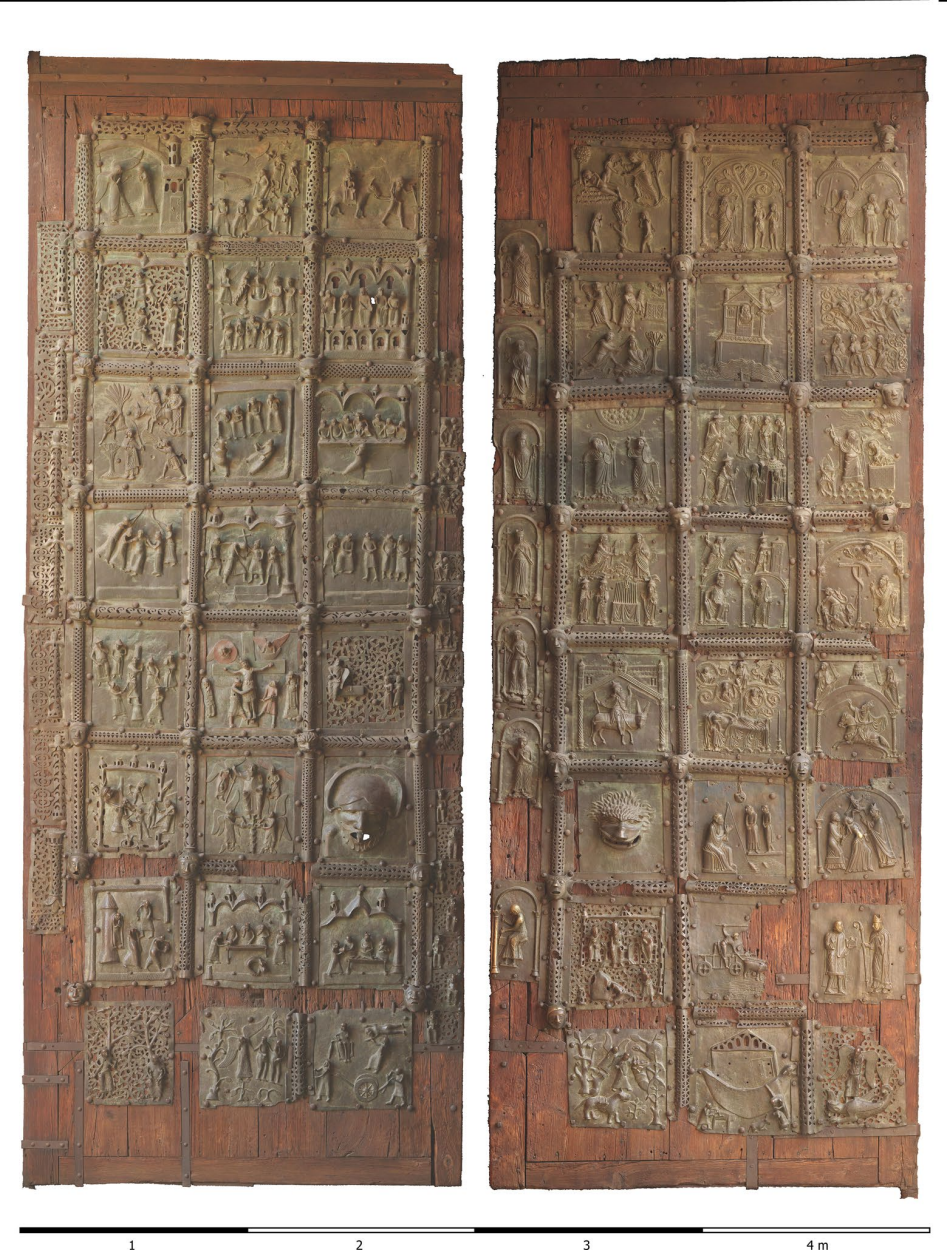
Orthophoto of the bronze doors from San Zeno, Verona, Italy

**Fig. 2:**
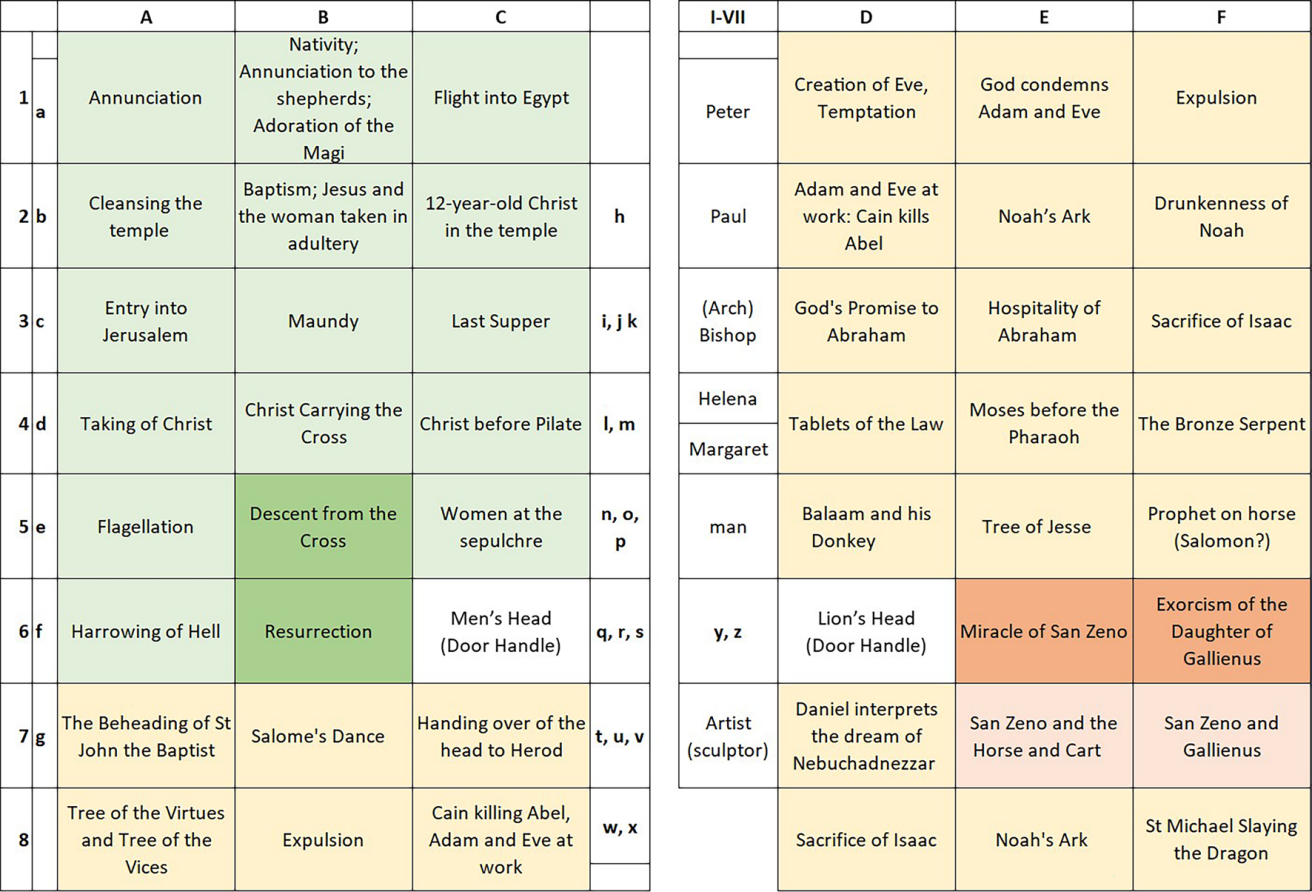
The bronze doors from San Zeno, Verona, Italy. Plate overview and numeration of the bronze doors. **a**–**g**: Ornamental panels each with a column and border ornamentation; **h**–**x**: small square plates (**h**–**n**: Enthroned; **o**: Temperantia; **p**: Justitia; **q**: Fortitudo); **r**–**z**: enthroned kings. The colours indicate scenes from the Old and New Testament (yellow and green, respectively) and the life of San Zeno (red)

**Fig. 3:**
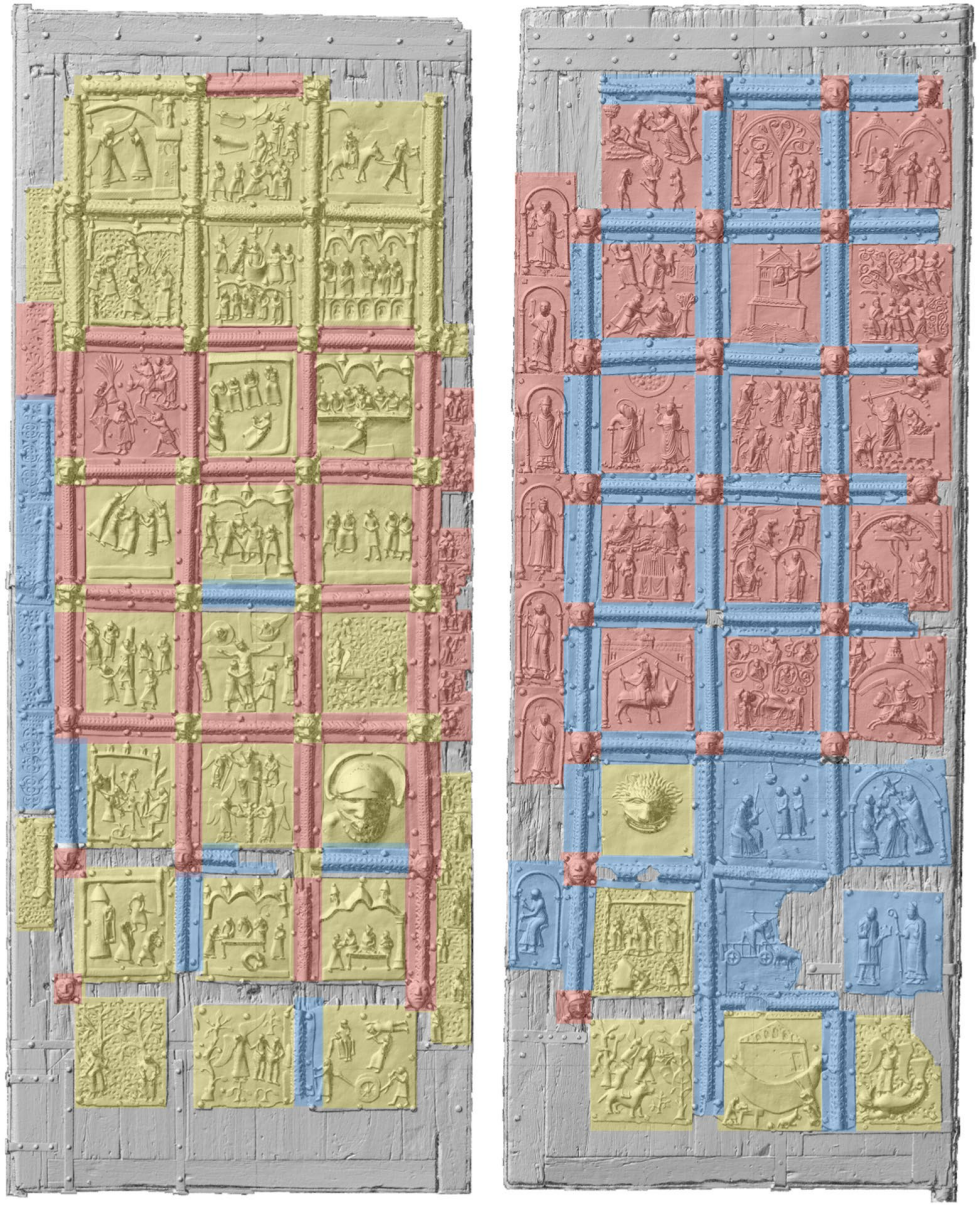
The attribution to different workshop of the single metal parts of the door according to [7]

The Bronze Door of Verona (Figs. 1 and 2) is a special case for art historical research. Several workshops must have produced the panels of the door at different times, but there is uncertainty about the dating of the various interventions [2–8]. Stylistically, it is possible to distinguish between two or even three workshops [7] (Fig. 3). In this study, our objective was to authenticate the art historical interpretation by determining the alloy composition of every individual metal plate.

According to stylistic interpretation, an older workshop (A) is responsible for 19 scenes from the New Testament and 5 from the Old Testament on the left wing. It also produced 8 of the 19 smaller panels with kings playing musical instruments and 12 fragmentary panels with openwork columns [7]. The narrative panels are characterised by a greater number of figures, most of which are positioned freely in the picture space. The robes have been reworked in detail using punching and hatching. In some panels the background is completely dominated by pierced vines. There are analogies here with other bronze objects that may have come from the same workshop, such as the fragment of the seven-branched candelabra in Klosterneuburg Abbey, and a small box in the *Museum für Kunst und Gewerbe* in Hamburg [1]. Moreover, there are stylistic and formal parallels with the door of Płock, now in Novgorod, which was made in Magdeburg in the mid-twelfth century. However, it is also possible that the two doors had common models [1]. This also raises the question of whether this first workshop (A) was based in Verona at all. The panels could have been imported to Verona, as was the case with Byzantine doors in Italy and some other Italian doors (such as those of Barisanus of Trani and Bonanus of Pisa in Monreale). The Veronese panels have an average size of 41 × 46 cm (± 2 cm) and are between one and a few mm thick, so transporting them was not difficult.

The majority of the panels on the right wing are stylistically very different from those described above. They appear more 'elegant' and 'natural', probably due to the scenic and architectural setting of the figures, which no longer float freely in the pictorial space [1]. This second workshop (B) produced 15 Old Testament scenes, as well as several centrally arranged rectangular panels depicting Saints and bishops (Fig. 3). It is disputed whether the four scenes from the Vita of St Zeno and another central panel, probably a self-portrait of the artist, can be attributed to a third master (workshop B/C), or if these panels are also produced by workshop B. It is also possible that several hands were involved in making the wax models for the plates in this second workshop (B) [1, 2].

It is unclear when the two or three workshops were active. Boeckler argues convincingly that the first workshop (A) was established around 1100 or shortly thereafter [2, 3] the first workshop (A) produced five scenes from the Old Testament in addition to the New Testament panels, it is clear that other panels with scenes from the Old Testament must have existed originally. It is possible that the two biblical cycles formed the two wings of a complete door [7]. This is also supported by the fact that both door pulls can be attributed to the first workshop (A). Some event must have led to the destruction of part of the door and with it, the bronze panels covering it.

Recent literature has suggested that the destructions of some panels may have been caused by the earthquake of 3 January 1117 which destroyed large parts of Verona [8, 9]. A possibly local bronze workshop may then have produced the Old Testament scenes. The fact that this second workshop complemented a pre-existing door is evident given the identical dimensions of the panels from the first and second workshops. On stylistic grounds, it has often been argued that the wax models of the second workshop may have come from the workshop of the sculptor Nicholas, who made the western sculptural portal of Verona Cathedral in 1139 and the reliefs that frame the bronze portal of San Zeno [4, 10, 11]. In this context, the artist's self-portrait was interpreted as a sculptor working in a stonemason’s workshop [4]

Another possible reason for the existence of two separate workshops (A and B) in Verona could be the restoration of the basilica at the end of the twelfth century. The old door that adorned the portal may have been too small after the alterations [4, 5, 7]. It has been also suggested that the second workshop (B) panels date from the late 12th or early 13th century, when the architect Briolotus carried out further work on the façade [2, 3, 5]

Besides the unclear dating of the two workshops, other open questions remain. The Old Testament scenes are to be understood as complementary to the New Testament scenes of the left wing. The narrative panels have the same dimensions, and the second workshop (B) was stylistically inspired by the first (A) [7]. Thus the duplication of some scenes (Expulsion from Paradise, Cain and Abel, Sacrifice of Isaac) is surprising. This cannot be explained by the theory that the panels of the first workshop (A) were supplemented by those of the second (B).

Moreover, it is not even clear whether the panels of the first workshop were actually made for San Zeno, or whether they were previously located elsewhere in Verona. The door must have been moved to its present location at the latest with the production and positioning of the four scenes of the Zeno legend.

Technologically, the two or three workshops are indistinguishable. All plates and frames were made using the lost wax technique. This means that a wax model (usually beeswax, or beeswax with various additives to alter its mouldability and stability) of the entire plate was made, embedded in clay—usually in several layers of decreasing fineness—and then heated. This removed the wax, baked the mould and allowed the liquid metal to be poured in. When the metal had cooled, the mould was destroyed and the surface was worked over using various tools. The finished plates, frames and heads were then fixed on the wooden base of the door. For this, over 650 nails of ten different types and shapes (made only of iron, or of iron with a copper alloy head) were used over the course of time [2, 7].As the plates have been removed several times over time, it is difficult to determine whether the nails (and plates) have always been returned to their original position.

In brief, our objective is to support, in one way or another, the art historical interpretation of two or even three different workshops that worked on the metal doors of San Zeno, Verona. We also investigate the wooden beam to determine its medieval origin and its potential contribution to the visual appeal of the door to medieval spectators.

## State of the art

As indicated by an article in the Italian newspaper L’Arena from august 1983, chemical analyses on the metal plates were carried out during restoration works by the *Opificio delle pietre dure*, Florence, Italy and the Institute of applied Chemistry, Rome, Italy. However, the results of analyses were never published. Further chemical and metallographic analyses of four selected plates (F4, E7, k and q) were carried out by Leoni [12]; further analyses were mentioned [2, 7]. Boeckler for instance mentions chemical analyses of unknown type of the today lost plates from the former *Kaiser-Friedrich-Museum*, which are associated with workshop A (Cu: 90.2 wt.%; 7 wt.% Sn; 2.8 wt.% Pb; 0.06 wt.% Fe). The microstructures published by Leoni [12] indicate an as-cast and slightly annealed microstructure with, in part, heavy deformation on the surface (slip lines) due to chiselling. These analyses provided the first indications that the workshops used different alloys (leaded bronze by workshop A (represented by plate q) and leaded brass by the workshops B and B/C, represented by plates F4, E7 and k). Unfortunately, the source of the copper ore cannot be determined by lead isotope analysis because significant amounts of Pb were added to the alloy, effectively overwriting any indication of Pb isotopes derived from the copper ores.

As far as the wooden base of the doors is concerned, in 1984 the boards were subjected to scientific tests to check for the presence of decaying agents, to identify the species of wood used and to date the wood using the technique of dendrochronology. The results were never published, apart from a few details [13].

## Methodology

### Photographic documentation

For the documentation of the preceding analyses, a comprehensive photographic record of the doors was created, using a methodological framework and workflow already applied at the bronze doors of San Marco, Venice [14]. The primary objective was to establish a precise measurement foundation by capturing exact points of measurement, encompassing both the geometric features and surface textures in high detail. Apart from capturing high-resolution images of individual panels, the individual door leaves were also documented using image-based modelling techniques.

Image-based modelling involves utilizing photogrammetry to reconstruct three-dimensional surfaces from a series of 2D photographs taken from various camera positions [15]. This technique has found increasing application in fields like archaeology and art history, offering the capability to generate highly accurate and detailed digital models of cultural heritage items, architectural structures, and artefacts. Beside the use for research and scholarly purposes its utility shines particularly in safeguarding and documenting delicate or hard-to-reach objects, and in creating virtual exhibitions and educational resources [16, 17].

The choice of an image-based approach is based on the primary objectives of the documentation to provide a metric basis for the mapping of the metallurgical analysis. While other methods, such as laser scanning or structured light scanning, can provide high quality and high-resolution results [18], their operation, handling and cost can be prohibitive. The method and equipment’s scalability and robustness enabled the smooth integration of photographic documentation alongside other data collection steps. The documentation work was carried out while the scaffolding provided access to the upper parts of the door, therefore the individual leaves were captured in their open positions as separate components. This data acquisition took place concurrently with chemical analyses and wooden structure observations. The images were captured using a Ricoh GR IIIx camera, featuring a 26.1 mm focal length (57° diagonal angle of view) and an RGB primary colour CMOS sensor (23.5 mm × 15.6 mm, 24.24 megapixels, pixel pitch 3.9 µm). The camera was handheld and used in separate acquisition steps for different parts of the door on the scaffold for the upper and ground based for the lower parts. Illumination was provided by the natural light, avoiding direct sunlight on the object. The processing was performed using *Agisoft Metashape* software (Version 1.7.2). Scale was established through measurements of distinct distances on the object and the inclusion of scaled poles in the acquired photographs, which were employed as reference scale bars during processing.

The resulting data set for the right and the left wing of the door (left: maximum dimensions 4.98 × 1.9 m, coverage area 9.38 m^2^; right: 4.97 × 1.92, 9.52 m^2^) was generated through 701/782 images with an average perpendicular distance of 1.25/1.13 m. The overlap exceeded 60%, and most areas were captured by at least 8 images. The outcome was a Digital Surface Model (DSM) with a resolution of 0.18/0.16 mm/pix. This served as the basis for generating a true orthophoto as an orthomosaic with a resolution of 0.2 mm/pix (Fig. 1).

Further stages of processing involved creating a textured 3D model derived from a point cloud containing 30 million points for each of the leaves. Due to spatial limitations, a separate model was generated for each door wing. The backside and technical details of the door were documented through regular photographs.

**Fig. 4:**
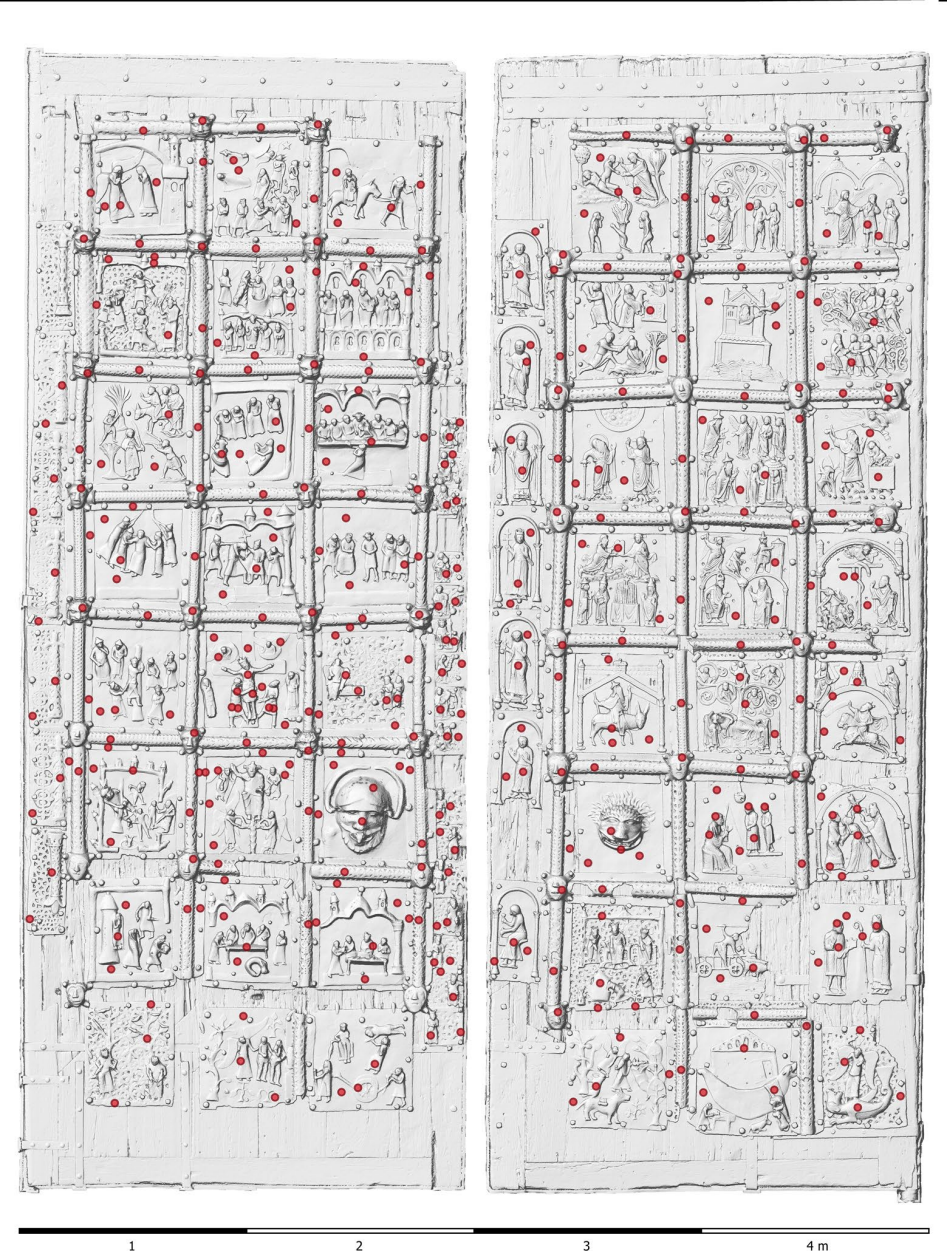
The bronze doors from San Zeno, Verona, Italy with indicated localisation of measurement points for XRF analyses

**Fig. 5:**
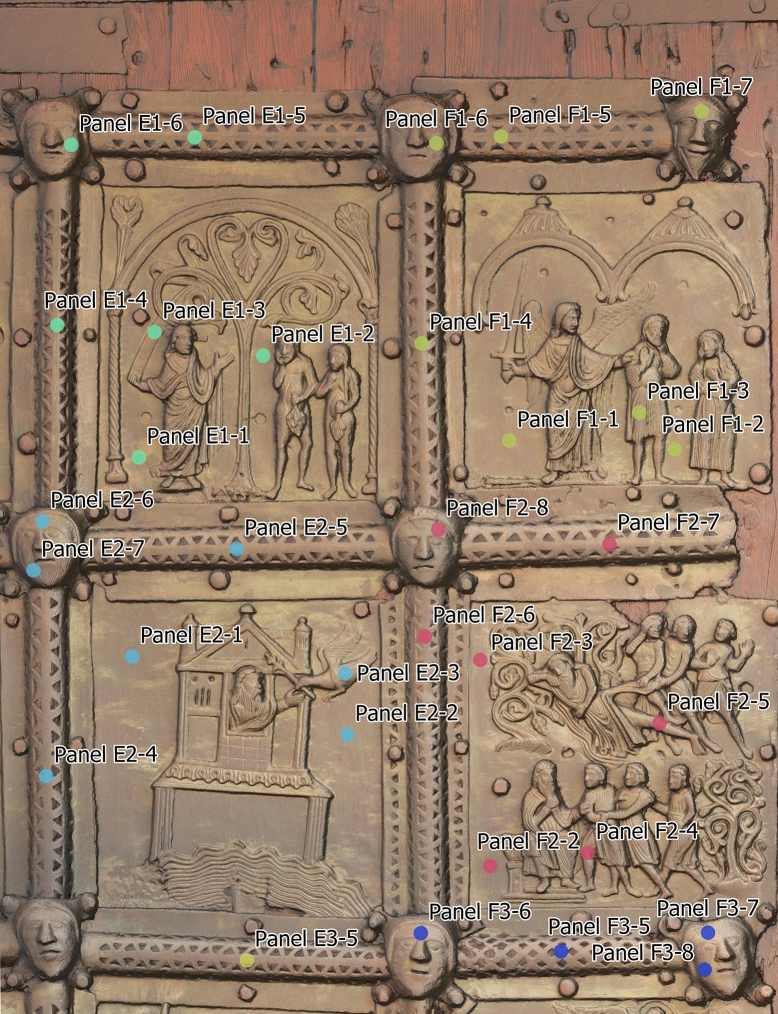
Detail of Fig. 4. Depicted are the panels E1, F1, E2 and F2

The comprehensive dataset accrued during our investigation was managed in a geospatial database within a desktop Geographic Information System (GIS) environment. This amalgamated dataset encompasses tabular data originating from chemical analyses, geometric data derived from three-dimensional (3D) modelling, and detailed images of the individual doors, focussing on artistic and construction details of the doors. These data sets were systematically organized and visualized for analytical purposes (Figs. 4 and 5).

These data assets empower us to undertake comparative studies extending beyond individual object elements. Such investigations can support the analysis of intricate details pertaining to production processes and interrelationships indicative of specific workshops.

### Chemical analyses and principal component analysis (PCA)

A portable ED-XRF (energy dispersive X-ray fluorescence) analyzer from Oxford Instruments was used to determine the chemical composition of the metal parts (model: X-MET5100, equipped with a high resolution detector 45 kV Rh target X-ray tube with max. 50 µA; measurement conditions: 40 kV voltage, 10 µA current and 60 s acquisition time). Spot measurements are approximately 9 mm in diameter. It is worth noting that ED-XRF measurements on corroded metals can give biased results if the penetration of the corrosion layer is deep enough (typically from 50 μm depth), making it difficult to distinguish between elemental contributions from the bulk and those from the patina [19–21]. However, according to [22], reliable results for major elements can be obtained when the patina is less than 25 μm deep. Based on [12], we can estimate an average corrosion penetration depth below this value, making the ED-XRF measurements reliable. Alloying elements such as Cu, Sn, Zn and Pb were detected quantitatively, while other elements were only detected qualitatively due to the presence of surface corrosion layers [23, 24]. S is not detectable by this ED-XRF. Alloys of different chemical composition produced by the authors were used as calibration standards [14] also to evaluate accuracy. Each metal plate of the doors was analysed on 5–10 different areas, allowing a mean chemical composition of each plate to be obtained (Figs. 4 and 5). Additional analyses were performed on the coloured areas of the four plates B5, B6, E6 and F6.

A Principal Component Analysis (PCA) was conducted on the data matrix following a protocol described elsewhere [14]. Briefly, the purpose of this procedure is to extract the maximum amount of information from the multivariate data structure by representing it as linear combinations of the variables. Principal Component Analysis (PCA) involves rotating the original dataset into a new geometric space. In this new space, the x-axis represents the first principal component (PC1), and its orientation aligns with the direction of maximum variance in the data. The y-axis, representing the second principal component (PC2), is perpendicular to PC1 and aligned with the next highest variance in the data. This rotation allows for a meaningful representation of the dataset in terms of its maximum variance. PCA generates two graphical representations: (i) loadings and (ii) scores. The loadings are depicted as a matrix, where the columns represent the eigenvectors and the rows represent the original variables. Each row contains numerical coefficients that indicate the significance of each original variable within that eigenvector. On the other hand, the scores are presented as a matrix in which the rows represent the samples, and the columns represent the principal components. The scores matrix displays the projections of the samples in the newly defined space. The calculation on the raw data, which was centered and autoscaled, was carried out using CAT (Chemometric Agile Tool) software [25].

### Wood analyses

No micro-invasive sampling was allowed on the wooden structures of the San Zeno doors. We therefore carried out observations (approximately 60 points) on the back of both wings using the Dino-Lite portable microscope to: (1) confirm the wood species as they are known; (2) identify any possible presence of biotic degradation agents. The Dino-Lite used, connected directly to a laptop, has a resolution of 2592 × 1944 pixels and a magnification of 200 ×, allowing the main anatomical features of the wood to be assessed and the species to be identified thanks to the comparison with the main atlas of wood anatomy [26, 27].

## Results and discussion

### Photographic documentation

The strategy for the photographic and image-based modelling documentation in our project has yielded substantial benefits across multiple dimensions [14]. The orthophoto with the mapped measurement points played a pivotal role in supporting the spatial documentation of chemical analyses, facilitating the interpretation of the constructions of the artworks, and concurrently offering insights into the current conservation status of the studied artifacts.

In alignment with the imperative of long-term data preservation, we are currently preparing the dataset for deposit into the IMAREAL repository, situated at the University of Salzburg. This preparatory phase entails the incorporation of meta- and paradata as of semantic annotations, underpinned by the well-established ontology of the RealOnline database. This strategy serves to enhance the dataset’s accessibility, opening avenues for future research endeavours. The enhanced accessibility of high resolution orthoimages, associated high resolution photographs of technical details and visualisations of digital surface models facilitates collaborative research initiatives while additionally serving as the cornerstone for a utilisation in the conceptual framework of Heritage Digital Twins. This innovative concept has recently emerged in the field [28].

### Chemical analyses and PCA

#### Alloy composition

Due to the abundance of raw data, a statistical approach was followed, and a principal component analysis (PCA) was computed. The raw ED-XRF data are arranged in matrix composed of 407 rows and 8 columns, corresponding to the sites of analysis (samples) and the elemental composition (variables), respectively (Additional file 2: Table S1, raw data with instrumental standard deviation). The entire set of data are points with new coordinates in the new orthogonal space created by the principal components (score plot). Based on the variance–covariance results extracted from the PCA analyses the first two PCs were chosen (PC1 and PC2), whose sum expresses the 57.74% of the explained variance. It is worth mentioning that this is a preliminary scrutiny of the raw data and considers all the elements composing the door, namely the plates (main square plates; side decorative plates; small square plates; plates depicting different personalities), the decorative frame elements and the heads. Additional file 1: Fig. S1 describes the biplot plot where the samples are projected in the new orthogonal space based on their composition (correlation to the alloying elements). As observed, most of the ED-XRF measurements are centred around 0, forming the main cluster. Some outliers are also discerned, some corresponding to the coloured plates (B5, B6, E6 and F6), which clearly have a prevalence of Pb and As due to the presence of pigments as minium/Pb_3_O_4_, Realgar/AsS, As_2_S_2_ or As_4_S_4_ cinnabar/HgS (Additional file 2: Table S1, see below).

**Fig. 6:**
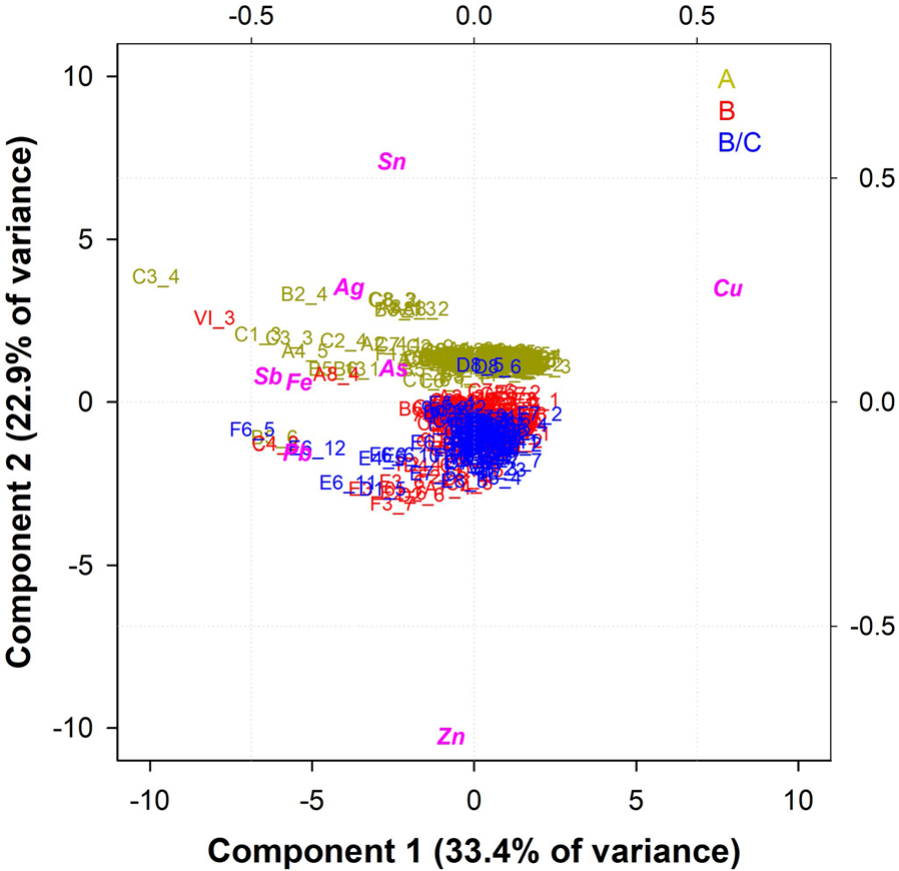
Biplot plot of the ED-XRF measurements, ruling out the coloured areas

After eliminating the points that do not provide a reliable interpretation of the door composition, a new PCA was performed and the graph in Fig. 6 shows the biplot of the results obtained for each ornamental panel (from A to F and from I to VI), decorative elements (from a to z), frames and heads. The first two components, having a good explained variance (33.4% for PC1, 22.9% for PC2), were chosen for the analysis. As visible from the biplot, two main clusters can be distinguished and attributed to two different types of bronzes. The upper cluster (yellow) is richer in Sn while the lower one (red and blue) is richer in Zn. Both clusters show some variations in the Pb supplementations (as displayed by the distribution of some points along the x axis). Some points far from the clusters are also observed. The measurements falling in the upper left part of the graph (corresponding to A4_5, B2_4, B6_1, C1_3, C2_4, C3_3, C3_4, taken from areas in relief) show a high Fe content, which can be due to a contamination from iron nails washed out by water and redeposited on the surface of the door. In addition, point VI_3 cannot be considered a reliable result due to the higher amount of Pb, Sn and Sb, which is not compatible with the other points of the same plate. These data can be related to a probable provisional repair (possibly using a Pb based alloy), as the casting quality of the panel in the area analysed is considerably poor. Furthermore, in the lower left of the graph, some points (B5_6, C4_2, E6_12, F6_5) are characterised by a high Pb content (19–32 wt.%), probably due to segregation during the cooling of the alloy after casting. This is confirmed by a slight downward shift of the cluster, confirming the heterogeneity of the alloy composition. These differences in chemical composition between different measurements of the same plate can be explained by (1) the segregation of the alloy during cooling; (2) the presence of corrosion layers affecting the measurement; (3) the penetration depth characteristics of the XRF instrument.

**Fig. 7:**
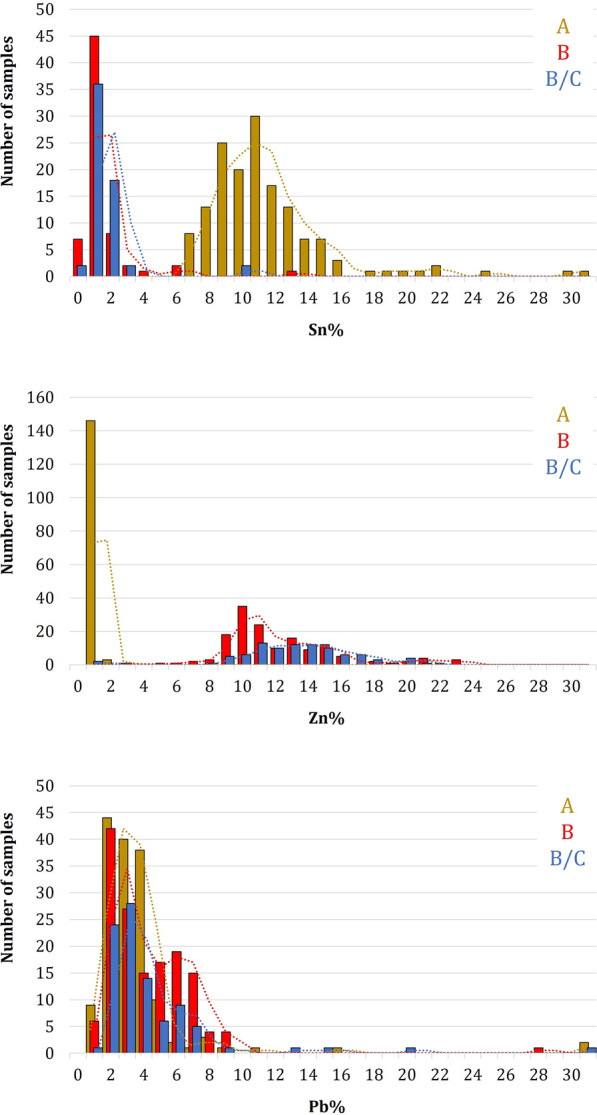
Frequency distribution of the main alloying elements according to the workshop categorization (workshop A: 152 points; workshop B: 149 points; workshop B/C: 91 points): **a** Sn; **b** Zinc; **c** Pb

The analysis confirms the usage of two different types of alloys, which coincide with the historical documentation that attributes the panels of the upper cluster to workshop A while the panels of the lower cluster are attributed to workshops B and B/C [7]. The alloy composition of the first cluster, which corresponds to the stylistical classification of workshop A, is richer in Sn, with the element in the alloy varying from 7 to 16 wt.%. Small amounts of Zn (around 1 wt. %) and a Pb from 2 to 4 wt. % make it a leaded bronze (Fig. 7). Similarly, the second cluster, corresponding to workshops B and B/C shows a Zinc content between 8 and 20 wt. %, a Pb content of 2–4 wt. % and a Sn below 3 wt. %, which gives an alloy composition typical of a leaded brass (Fig. 7). All the panels from workshop A have a similar chemical composition, regardless of whether they depict scenes from the Old or New Testament, except three (A8, B8, C8). In fact, a micro cluster can be observed in the upper part of the graph, including the points measured in panels A8, B8 and C8. It demonstrates that the composition of these panels is slightly different (higher contamination of Ag and Sb).

**Fig. 8:**
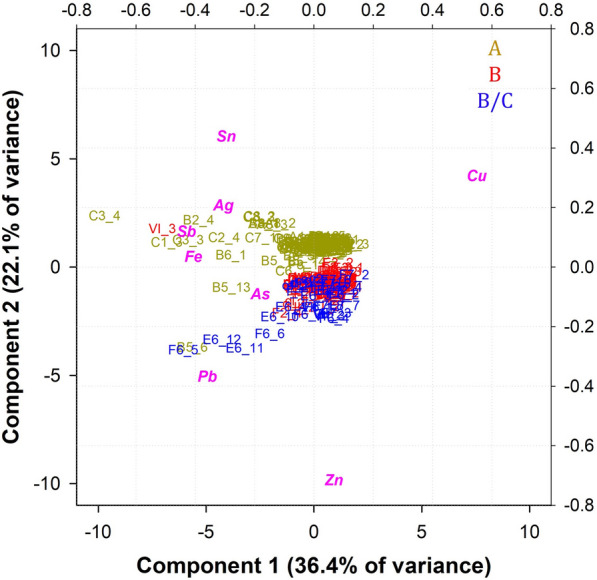
Biplot of the ED-XRF measurements on panels

To detail the alloy composition differences more thoroughly and possible anomalies in the historical attribution, PCA computations were performed on panels only, frames only and heads only. Figure 8 displays the PC1 vs PC2 biplot (explained variance of 54.5%) of the panels measurements.

This plot confirms the previous results, although with a less defined cluster difference: B2_4, B6_1, C1_3, C2_4, C3_3, VI_3 show Fe contamination from iron nails; B5_6, E6_12, F6_5 show high Pb content and panels A8, B8 and C8 are richer in Sn than the other panels of workshop A. A further discrimination of points with high Pb content is observed for measurements B5_13 and E6_11.

**Fig. 9:**
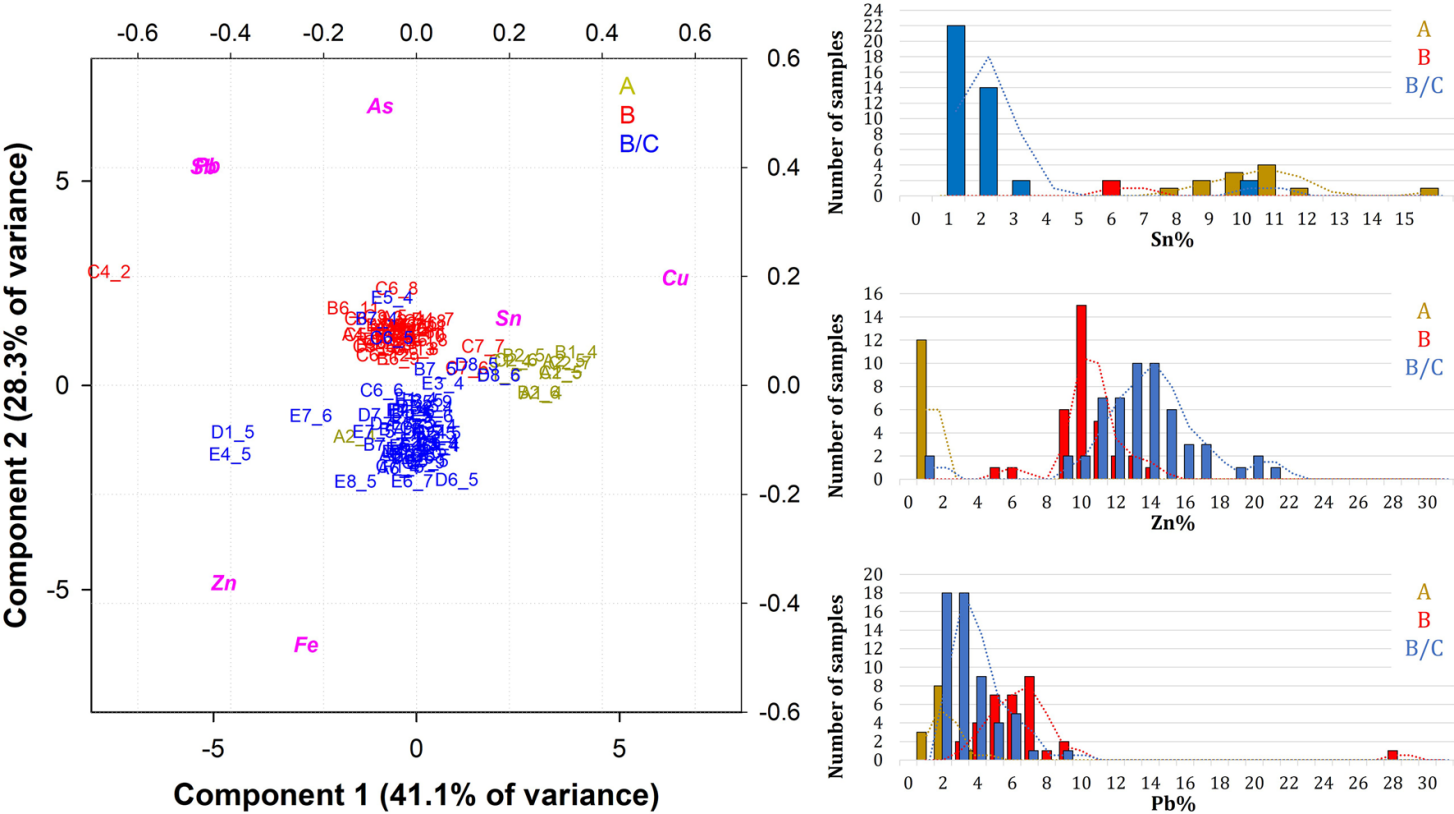
PCA on the decorative frames and frequency distribution of the main alloying elements according to the workshop attribution: **a** Biplot of the ED-XRF measurements on frames (workshop A: 11 points; workshop B: 33 points; workshop B/C: 56 points): **b** Sn; **c** Zinc; **d** Pb

No average (i.e. the average of several analyses of the same metal part) was taken into account for the ornamental frames, since only one or two measurements were made per frame. The biplot of PC1 vs. PC2 (total explained variance of 69.4%), visible in Fig. 9, more obviously supports the evolution of the composition of the alloys used to produce the decorative frames. Three clusters are clearly observed around the centre point (0) and are differentiated by the amount of alloying elements. Interestingly, this classification only partially agrees with the stylistic attributions of art historians (A, B and B/C) and excludes some frames, which are made of a different alloy, leaving out C4_2, already discussed.

These clusters are chemically distinct in terms of Cu, Sn and Zn content, in line with the overall results (use of a leaded bronze for workshop A and a leaded brass for workshops B and B/C). However, as visible from the plot, the blue cluster and the red cluster diverge along the y axis, where most of the variance of PC2 is explained by Sn. This implies that the Sn content is discriminant for two of the three clusters, which could indicate the use of two different alloys. Workshop B uses an Sn-free alloy, with the only exception of the right frame of C7. On the other hand, workshop B/C uses a leaded brass with small but substantial amounts of Sn (for the most part from 1 to 3 wt. %) as displayed also by the frequency plot of Fig. 9.

Furthermore, some decorative frames that stylistically fall into the art history attribution, chemically belong to a different cluster. Exceptions are discussed as follows (see Fig. 9):

1. The frame on the right of panel D8 (stylistically associated with workshop B or B/C) has a chemical composition typical of workshop A. The most likely explanation is that they are remelting parts from the other workshop—which would indicate the availability of alloy A for workshop B/C.
2. The frames to the left of panels B7 and E5, all of which are attributed to workshop B/C have the same chemical composition of workshop B. However, the alloy difference is marginal and only in the amount of Zn. In this case, we do not exclude the possibility that workshops B and B/C worked simultaneously.
3. The frame on the top left of C7 in terms of chemical composition is inconsistent with the average composition of its stylistically associated workshop (B); the chemical composition of the frame element is exactly between the yellow (workshop A) and the red cluster. It is made of a quaternary bronze (Sn 5 wt.%, Zn 5 wt.% and Pb 3 wt.%). Similarly, in the case of the frame of the C7 panel, we cannot exclude the possibility of partial availability for workshop B of pieces of alloy A remelted to make this frame.
4. The frame on the left of panel A2 (attributed to workshop A) seems to belong in terms of chemical composition to the blue cluster (workshop B/C). However, the contents of Zn, Sn and Pb are consistent with the average of workshop A, except for a very high Fe content (4 wt. %), likely related to iron nail contamination. Thus, the shift of this measure towards the Fe variable inevitably includes it in the blue cluster. This is related to the scaling step of the PCA, which normalises the data so that all variables are on the same scale. Thus, important elements are weighted as minor elements, but without their reliability; consequently, the frame on the left of panel A2 remains associated also chemically with workshop A.

**Fig. 10:**
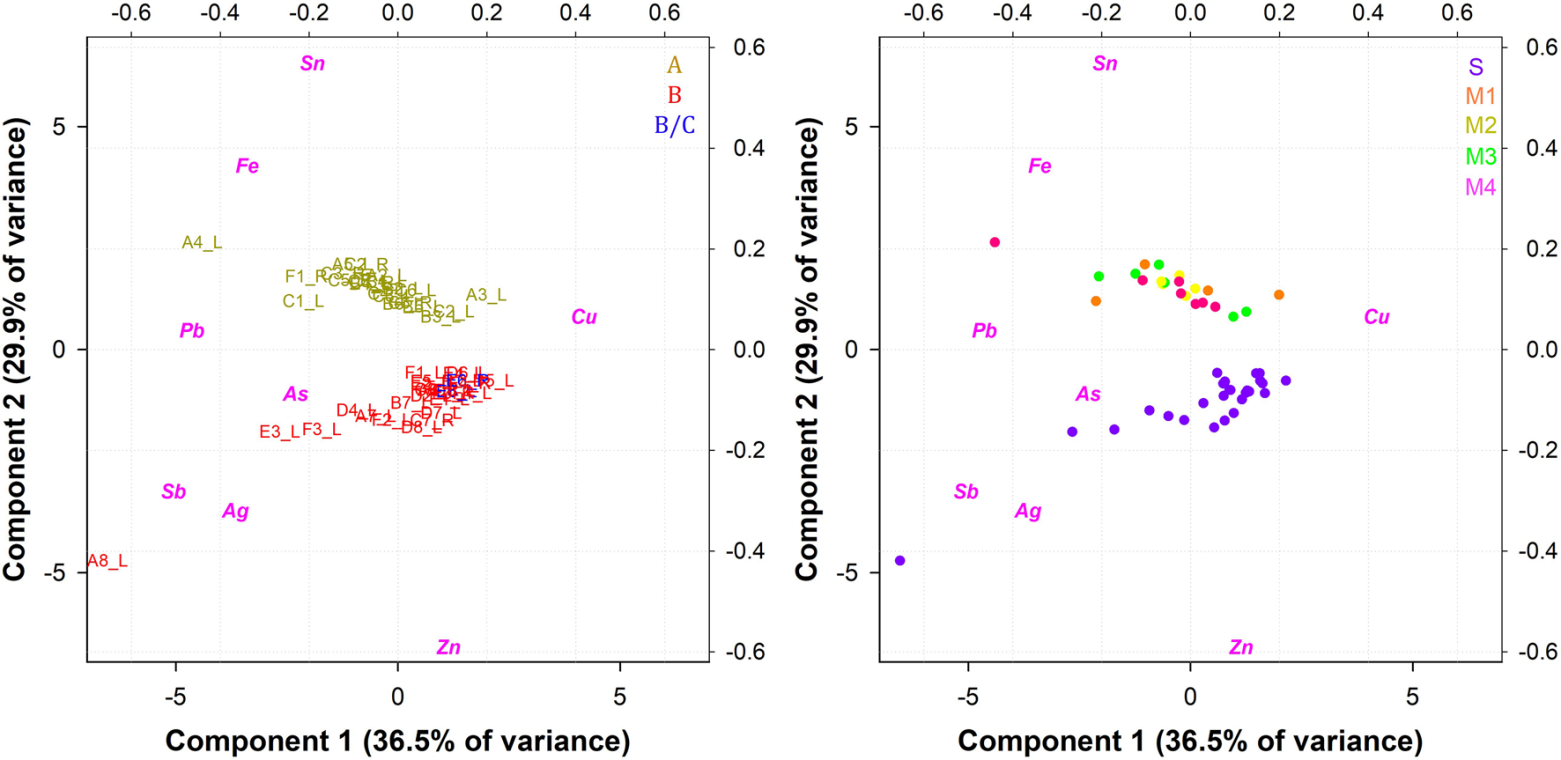
Biplot of the ED-XRF measurements on heads: **a** historical classification; **b** stylistical classification based on the use of different moulds (M). S = individual/single mould

Only one measurement was carried out on the heads. The resulting PCA computation (PC1 vs PC2 graph, total explained variance of 66.4%) is observed in Fig. 10a. As expected, the chemical distinction between the different workshops is well defined by the samples that distribute along two lines with different PC2 coordinates. Notably, only two heads are associated with workshop B1 according to art historical methodology; they match well the group of heads associated with workshop B2. The heads of workshop A were made of leaded bronze, while the heads of workshops B1 and B2 were made of leaded brass.

Moreover, the heads of workshops B and B/C are all single types; every head differs significantly from the others of the same workshop. On the opposite, four types of heads are confirmed for workshop A (Fig. 10b). Most likely, the same mould was used to produce each head of the same type. However, no specific chemical composition was noted for these types, a part for the head type M2; here, the chemical composition is basically the same. For M3, two different alloy groups are visible, related to the amount of Sn (an average of 10 wt.% vs. 15 wt.%), which might also be related to the presence of the rather thick corrosion layers of the heads.

#### Coloured plates

**Fig. 11:**
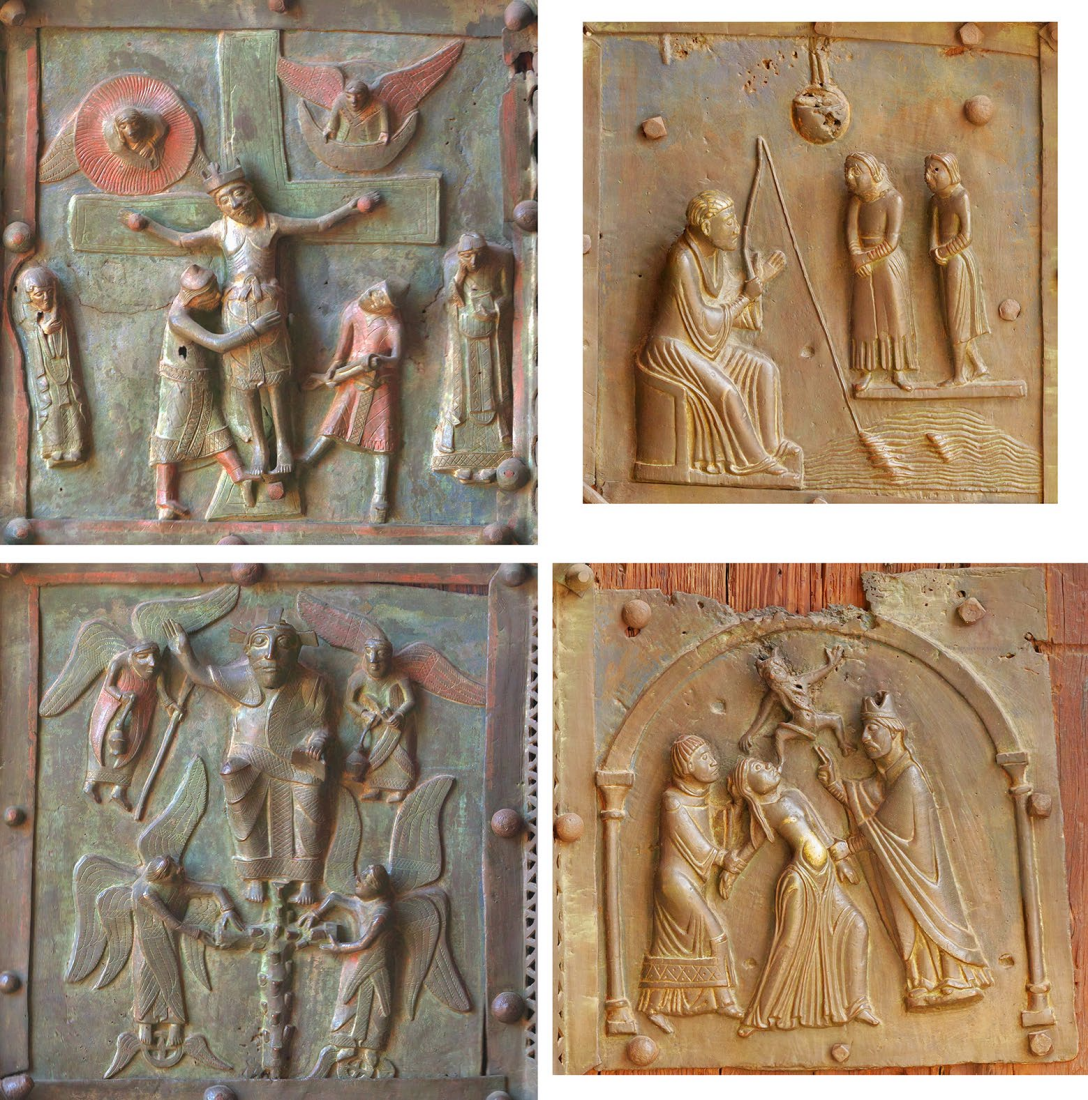
The coloured plates. Top left: B5; top right: E6; bottom left: B6; bottom right: F6

**Fig. 12:**
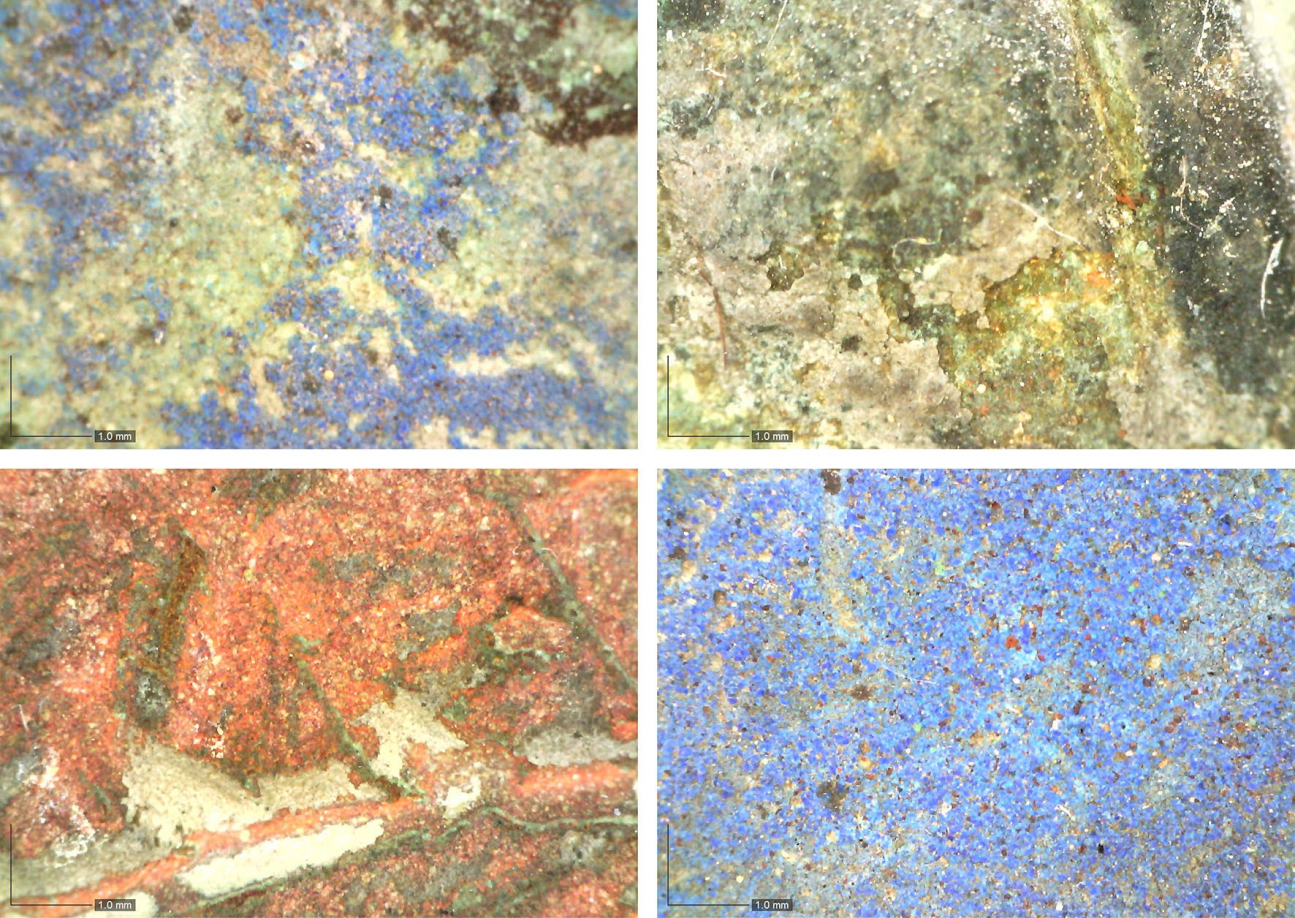
Details of the coloured plates B5, B6 and G6. Top left: B5, heaven; top right: B6, Jesus’ skirt (gilded); bottom left: B6, angel wing (cinnabar); bottom right: E6, heaven

Four of the metal plates, namely the two bronze plates B5 (Descent from the Cross) and B6 (Resurrection), as well as the brass plates E6 (Miracle of San Zeno) and F6 (Exorcism of the Daughter of Gallienus), show indications of artificial colouring. Residues from the red and blue colours are still visible today, while the former gilding was identified only by XRF and macro-imaging with a *Dino-Lite,* model AM7013MZT (Figs. 11 and 12). First analyses on the different colours of plates B5 and B6 (and their surrounding decorative elements) were already carried out at the beginning of the 1980s by the *Opificio delle pietre dure*, Florence, Italy and the Institute of applied Chemistry, Rome, Italy, but never published; an article in the local newspaper *l’Arena* from August 9, 1983, reports the identification of gilding, azurite [Cu_3_(CO_3_)_2_(OH)_2_] and cinnabar (HgS). These first results could be confirmed not only for the named two plates, but also for plates E6 and F6, where the colour blue for the upper areas of the background of the scenery.

Qualitative results allowing the identification of the staining products were obtained using ED-XRF, although S, a major component of cinnabar, cannot be detected by ED-XRF and the stained areas are not sufficiently well preserved for non-invasive quantitative analysis. Nevertheless, the following coloured regions were identified (Figs. 11 and 12):

1. Blue: likely heaven (plates B5, B6, E6, F6)
2. Gold: Jesus’ skirt and the golden belt of the soldier on the right (plate B5)
3. Red: angel wings and dresses (plates B5, B6), dress soldier right (plate B5), feet soldier left (plate B5), part of Jesus’ dress (plate B6), frame of the decorative elements around plates B5 and B6.

Areas decorated in green or yellow/ochre, as noted elsewhere [13], could not be identified. Moreover, the differences between cinnabar, cinnabar/ochre, red/lacquered, noted by the same authors, were no longer visible.

Blue areas—notable as background colour for the sky on all four coloured plates B5, B6, E6 and F6—show slightly higher quantities of Fe and of Sn (the latter concerning plates B5 and B6 only). To identify possibly used colouring or corrosion producing reagents, further analyses will have to be carried out, as the XRF analyses do not provide sufficient details for the identification.

The residues of the gilding (plate B5; Jesus’ skirt and the golden belt of the soldier on the right) indicate mechanical gilding. Here, it is also important to note that Jesus of plate B5 is a separate cast, which was mechanically fixed on his cross by iron nails. The 2.8 wt.% Hg reported in the analyses of the golden belt of the soldier are due to the size of the measurement area of the XRF, which also took into account the Hg-rich, red coloured skirt of the soldier.

The red areas were likely made of cinnabar, as indicated by the presence of Hg up to almost 17 wt.%; we have to take into account that S was not measured by the XRF. However, the measurements revealed also 26–38 wt.% Pb and about 1 wt.% As, which is significantly more than reported for the main alloys of both frames and decorative panels. Cinnabar is photosensitive and blackens when exposed to sunlight, as noted for instance on the walls of Herculaneum and Pompeii, but might be stabilized by using lead oxide (Pb_3_O_4_) [29]. Hence, the higher amounts of Pb in the reddish are likely not due to the usage of lead white [basic lead carbonate, 2PbCO_3_·Pb(OH)_2_], as this would have resulted first in a less bright red and later, as white lead is reduced by sulphides to black PbS, in a quite dark red, or reddish black [29].

Instead, the higher quantities of Pb and As point to a combined usage of cinnabar/vermillion (HgS), minimum (Pb_3_O_4_), and small quantities of Realgar (AsS, As_2_S_2_ or As_4_S_4_). Red ochre (Fe_2_O_3_) as potential additive is excluded, as no higher amounts of Fe were noted, as well as the usage of stibnite (Sb_2_S_3_), as we did not note any higher amounts of Sb. It has to be noted though that no indication about different layers of colour were observed; instead, the red has a quite uniform colour wherever preserved.

The presence of the red colour on the decorative frames around plates B5 and B6 (workshop A), which are associated with two different workshops (the frames above B5 and below B6 are stylistically related to the workshop B/C, while the others are related to the workshop B [7], indicates that the colouring of the plates was added after the production period of workshop A, at the earliest during the production period of the workshop B.

In addition to the deliberate colouring of at least four panels as described above, we must also take into account the different colours of the panels themselves: as they are largely made of leaded bronze (left wing) and leaded brass (right wing), it is likely that a difference in colour was also noted here. Colourimetric measurements are currently carried out on artificially produced alloy samples with a similar chemical composition and partially already published [30, 31].

### Wood analyses

**Fig. 13:**
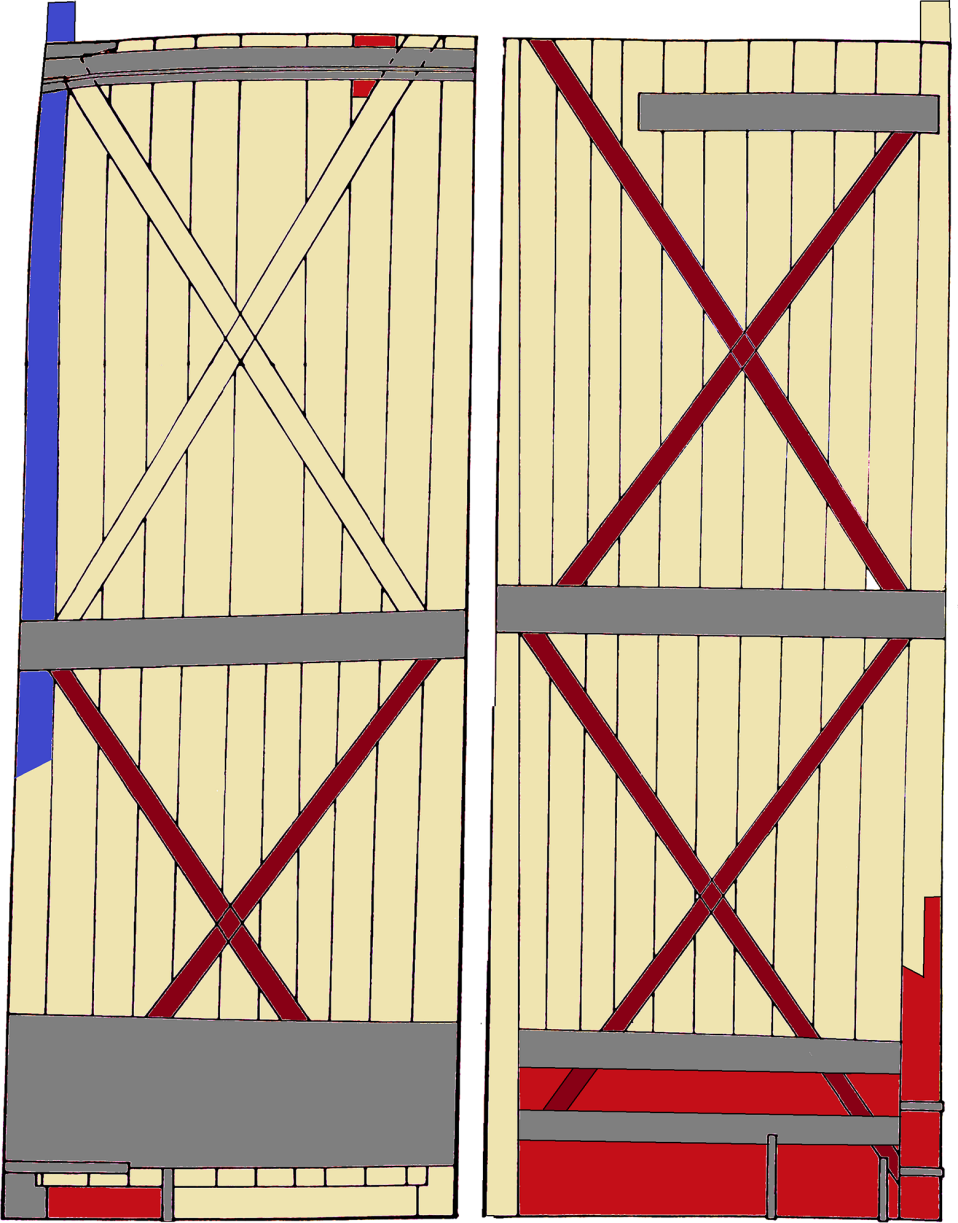
Diagram of the back of the door, in false colours. Spruce: yellow. Oak: brown. Pine: red. Yew: blue. The grey areas represent some of the many metal plates applied to the wood

**Fig. 14:**
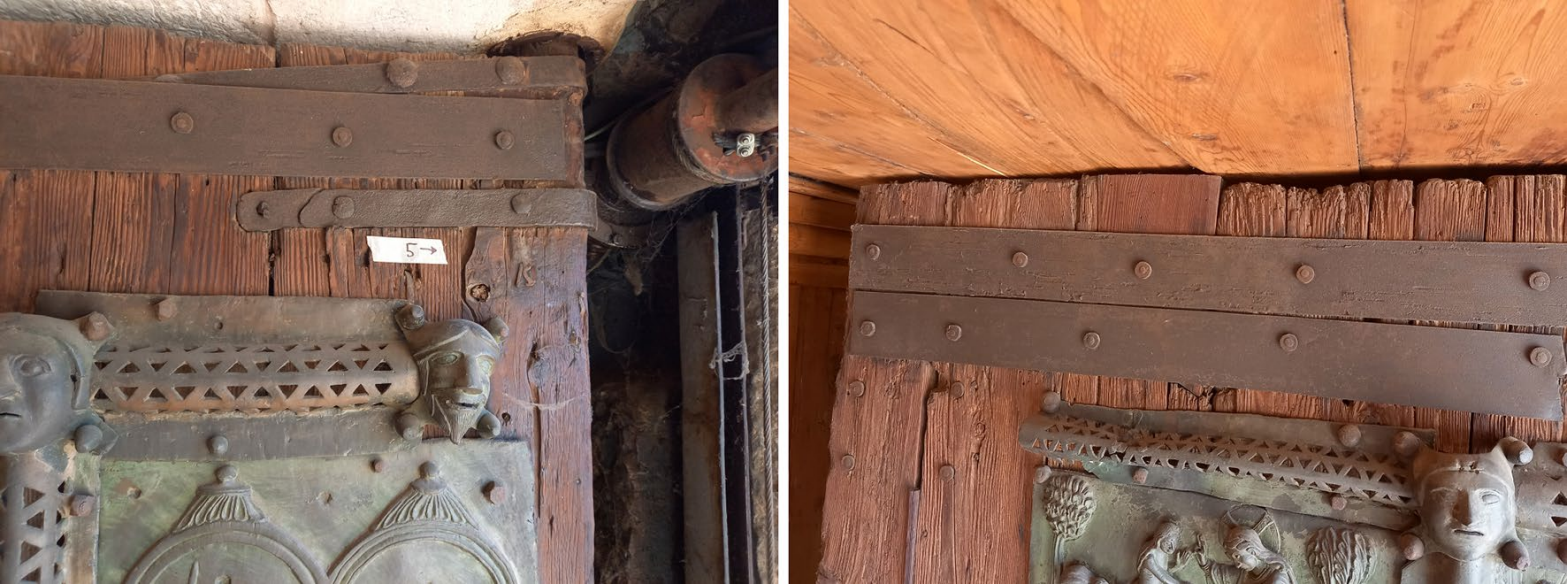
Details of the wooden door frame. Left. The upper corner of the right wing: you can see the vertical spruce boards and, at the end of the wing, where the arrow points, the board that also serves as the fulcrum. Right: vertical spruce boards and a part restored with pine wood

The non-invasive observations made with Dyno-Lite confirm the results of the extensive analysis carried out in 1984 and reported in the proceedings of the 1987 Trieste Conference [13]. The vertical thick boards are made of Norway spruce (*Picea abies* Karst.), while the bracings at the back of the door are mainly made of oak (*Quercus* spp.) (Fig. 13). Exceptions are the upper bracing system of the right wing, made of spruce, and the first board of the right wing, which serves as a pivot, made of yew (*Taxus baccata* L.) (Figs. 13 and 14).

This is a rather surprising result, considering that in Italy the yew trees are scarce, mostly found here and there in beech woods. However, the choice of this wood for a highly loaded element such as the door pivot is certainly strategic. Among the native conifers, yew is one of the densest woods with the best mechanical performance [32], as it has been the preferred one for making bows for over 5000 years (the scientific name of this species comes from the Greek word *toxon*, meaning ‘bow’). The upper bracing system of the right wing is made of spruce instead of oak, which is almost certainly the result of a reparation. Also small reparations, made of pine wood (*Pinus* spp.), are observed.

All the spruce boards are of the same age, as indicated by the dendrochronological study carried out in 1984 [13]. In the 1980ies, the wooden roof above the door was dismantled, which made it possible to observe the cross section of the vertical boards of the door. All the tree-ring curves obtained overlapped very well, confirming that all the spruce timbers of the door were made at the same time, not specifying the century. Unfortunately, in 1984, there were no well-replicated reference chronologies valid for spruce in the Eastern Alps, and consequently an accurate and reliable dating could not be obtained. As for the general condition of the wood, the Dyno-Lite digital microscope has shown that there is no ongoing biotic attack by insects on the wooden structures.

## Conclusion

In most cases, the chemical composition confirms the stylistical distinction between the two/three workshops. With regards to the open questions of chronological order and dating of the work of the different workshops, material analysis has not been able to make a significant contribution. Chemically, however, there is a clear distinction between Workshop A (tin-bronze) and Workshops B and B/C (brass). While the same alloy was used for the panels stylistically associated with workshops B and B/C, the situation is slightly different for the decorative frames stylistically associated with these workshops: workshop B uses a Sn-free alloy, while workshop B/C uses a leaded brass with Sn (1 to 3 wt. %).

The panels of the first workshop (A) can be dated to the first half of the twelfth century. They were possibly made before the earthquake of 1117 or partially destroyed during the rebuilding of San Zeno in the 1130 s. A second or even a third workshop (B, B/C) may then have been active in the course of these rebuilding measures (at the same time as the sculptor Nicholas) or only at the turn of the century around 1200. The place of manufacture of the older panels (A) is also unclear; the workshop could have come to Verona from the northern Alpine region, but it is also possible that the panels were imported. On the other hand, the later workshop(s) were most likely active in Verona itself, producing the San Zeno plates.

The most likely explanation for the observation that some of the plates and frames are stylistically associated with one workshop, but their chemical composition points to another workshop, is that they were re-melted from parts of the other workshop: workshop B and B/C remelted plates from workshop A.

We also tried to find any potential match between the chemical composition and the scenery depicted on the plates (Old Testament; New Testament; life of San Zeno), of which the latter matches roughly also the distinction between the different workshops. Only three plates, belonging to workshop A, show slightly different composition from the other plates of workshop A (higher Sb and Ag content): these plates depict scenes from the Old Testament and not, as most of the plates from workshop A, scenes from the New Testament. However, not all plates of workshop A, which are depicting scenes from the Old Testament, show such “contamination”.

Unfortunately, we could not determine if the wooden structure dates back to when the door was constructed. However, the wooden beam appears to have had a greater impact on the door's aesthetics compared to most similar doors, as it was visible through some of the perforated metal panels, such as those on window and door openings.

Four plates (workshops A and B/C) and some of the surrounding frames (from all workshops) show colouring (blue; cinnabar; gilding), which was added earliest during the work of the second workshop.

Concluding, we may state that we have discovered distinct styles and chemical compositions of the metal parts. It is unclear whether this indicates two different workshops, two different casters in the same workshop, or the same caster in the same workshop using different blends of alloy types.

Nevertheless, we may state that there were different artists (wax cutters) responsible for the production of the model.

Concerning the wooden support of the door, we could confirm that the door’s wooden base is made of spruce (vertical thick boards; upper bracing system of the right wing), oak (most of the bracings at the back of the door) and yew (the first board of the right wing, which serves as a pivot).

### Supplementary Information

**Additional file 1: Figure S1.** Biplot of the ED-XRF measurements

**Additional file 2: Table S1.** Chemical composition of the different metal parts of the bronze doors. Model 1. 3D-model of the left wing of the metal doors from San Zeno, Verona, Italy. https://sketchfab.com/3d-models/verona-san-zeno-maggiore-left-4abf577fd5d042e8b3af6d76780ef6ca. Model 2. 3D-model of the right wing of the metal doors from San Zeno, Verona, Italy. https://sketchfab.com/3d-models/verona-san-zeno-maggiore-right-9d1bd2889dcd450e8bc97e1d1cee7d5e.

## Data Availability

All relevant data are within the paper and its Supporting Information files.

## References

[1] Mende U. Die Bronzetüren des Mittelalters (The bronze doors of the Middle Ages): München; 1983.

[2] Boeckler A. Die Bronzetür von Verona (The bronze door of Verona), Marburg; 1931.

[3] Arslan E. La Pittura e la Scultura Veronese dal Secolo VIII al Secolo XIII (Veronese Painting and Sculpture from the 8th Century to the 13th Century). Milan: Fratelli; 1943.

[4] Fasanari R. I bronzi del portale di San Zeno (The bronzes of the San Zeno portal). Verona: Edizioni di “Vita veronese”; 1961.

[5] Zuliani F. La porta bronzea di S. Zeno a Verona (The bronze door of S. Zeno in Verona). In: Salomi S, editors. Le porte di bronzo dall’antichità al secolo XIII, atti del convegno internazionale di studi (Trieste, 13–18 aprile 1987). Roma: Istituto della Enciclopedia Italiana; 1990 (Acta Encyclopaedica, 15). p. 407–20.

[6] Mellini GL. I Maestri Dei Bronzi di San Zeno (The Masters of the Bronzes of San Zeno). Verona: Edizioni Bolis—Banca Popolare di Verona; 1992.

[7] Coden F, Franco T. San Zeno. Le porte bronze—the bronze doors. Cierre Edizioni: Caselle di Sommacampagna; 2017.

[8] Weinryb I (2016). The bronze object in the middle ages.

[9] Heginbotham C. The bronze door panels within the Façade of San Zeno Maggiore, Verona: a chronological and liturgical assessment. University of York; Master thesis. 2016.

[10] Olchawa J. Aquamanilien: Genese, Verbreitung und Bedeutung in islamischen und christlichen Zeremonien (Aquamanilia. Genesis, distribution and significance in Islamic and Christian ceremonies). Regensburg; 2019.

[11] Kain E (1981). An analysis of the marble reliefs on the façade of S. Zeno, Verona. Art Bull.

[12] Leoni M. Studio metallografico della porta bronzea della basilica di S. Zeno in Verona (Metallographic study of the bronze door of the basilica of S. Zeno in Verona). In: Salomi S, Editor. Le porte di bronzo dall’antichità al secolo XIII, atti del convegno internazionale di studi (Trieste, 13–18 aprile 1987), Roma, Istituto della Enciclopedia Italiana; 1990 (Acta Encyclopaedica, 15). p. 431–5.

[13] Aliberti Gaudioso FM, Pietropoli, F. Dendrocronologia dei supporti lignei e analisi dei materiali della porta bronzea di S. Zeno a Verona. In: Salomi S, editor. Le porte di bronzo dall’antichità al secolo XIII, atti del convegno internazionale di studi (Trieste, 13–18 aprile 1987), Roma, Istituto della Enciclopedia Italiana; 1990 (Acta Encyclopaedica, 15). p. 421–9.

[14] Mödlinger M, Bernabei M, Bontadi J, Fellin M, Fera M, Ghiara G (2013). Multidisciplinary analyses on the 11^th^–12th century bronze doors of San Marco, Venice. PLoS ONE.

[15] Remondino F, El-Hakim S (2006). Image-based 3D modelling: a review. Photogram Rec.

[16] Agnello F, Lo Brutto M, Lo Meo G (2005). DSM and digital orthophotos in cultural heritage documentation. Int Arch Photogramm Remote Sens Spatial Inf Sci.

[17] Agosto E, Bornaz L (2017). 3D models in cultural heritage: approaches for their creation and use. Int J of Comput Methods Heritage Sci.

[18] Hassani F (2015). Documentation of cultural heritage; techniques, potentials, and constraints. Int Arch Photogramm Remote Sens Spatial Inf Sci.

[19] Shugar AN, Mass JL, Shugar AN, Mass JL (2012). Handheld XRF for art and archaeology. Studies in archaeological sciences 3.

[20] Nørgaard HN (2017). Portable XRF on prehistoric bronze artefacts: limitations and use for the detection of bronze age metal workshops. Open Archaeol.

[21] Potts PJ, West M (2008). Portable X-ray fluorescence spectrometry capabilities for in situ analysis.

[22] Šatović D, Desnica V, Fazinić S (2013). Use of portable X-ray fluorescence instrument for bulk alloy analysis on low corroded indoor bronzes. Spectrochim Acta B: Atomic Spectrosc.

[23] Heginbotham A, Bezur A, Bouchard M, Davis J M, Eremin K, Frantz JH, et al. An evaluation of inter-laboratory reproducibility for quantitative XRF of historic copper alloys. In: Mardikian P, Chemello C, Watters C, Hull P, editors. Metal 2010: International Conference on Metal Conservation, Interim Meeting of the International Council of Museums Committee for Conservation Metal Working Group, October 11–15, 2010, Charleston, South Carolina, USA. Clemson: Clemson University; 2010, p. 178–88. https://repository.si.edu/handle/10088/90692. Accessed 3 Dec 2023.

[24] Heginbotham A, Bassett J, Bourgarit D, Eveleigh C, Glinsman L, Hook D (2015). The copper CHARM set: a new set of certified reference materials for the standardization of quantitative X-ray fluorescence analysis of heritage copper alloys. Archaeometry.

[25] Leardi R, Melzi C, Polotti G. CAT (Chemometric Agile Tool). http://gruppochemiometria.it/index.php/software/. Accessed 1 Oct 2023.

[26] Schweingruber FH (1990). The anatomy of European woods.

[27] Ruffinatto F, Crivellaro A (2019). Atlas of macroscopic wood Identification with a special focus on timbers used in Europe and CITES-listed species.

[28] Niccolucci F, Felicetti A, Hermon S (2022). Populating the data space for cultural heritage with heritage digital twins. Data.

[29] Nöller R (2015). Cinnabar reviewed: characterization of the red pigment and its reactions. Stud Conserv.

[30] Mödlinger M, Kuijpers M, Braekmans D, Berger D (2017). Quantitative comparisons of the color of CuAs, CuSn, CuNi, and CuSb alloys. J Archaeol Sci.

[31] Vernet J. Out-of-equilibrium behaviour of copper-based alloys in industrial, artistic and historical gravity casting processes, PhD thesis, University of Genoa, 2018. https://iris.unige.it/retrieve/e268c4c9-6c73-a6b7-e053-3a05fe0adea1/phdunige_3891483.pdf. Accessed 7 Dec 2023.

[32] Keunecke D, Märki C, Niemz P (2007). Structural and mechanical properties of yew wood. Wood Res.

