# Supplementary material for: The medieval bronze doors of San Zeno, Verona: combining material analyses and art history
Source: Herit Sci. 2024 Jan 25;12(1):26. doi: 10.1186/s40494-024-01143-2 (PMC10811183; doi:10.1186/s40494-024-01143-2)

**Additional file for**

# The medieval Bronze Doors of San Zeno, Verona: combining material analyses and art history

M. Mödlinger^1^, J. Bontadi^2^, M. Fellin^2^, M. Fera^3^, M. Negri^2^, J. Utz^1^, G. Ghiara^1, 4^

^1^ IMAREAL, Paris Lodron University Salzburg, Körnermarkt 13, 3500 Krems/Donau, Austria

^2^ CNR-IBE, Consiglio Nazionale delle Ricerche, Istituto per la Bioeconomia, via Francesco Biasi 75, 38098 San Michele all’Adige, Italy

^3^ Universität Wien, Institut für Urgeschichte und Historische Archäologie, Franz-Klein-Gasse 1, 1190 Vienna, Austria

^4^ DISAT, Politecnico di Torino, Corso Duca degli Abruzzi 24, 10129 Torino, Italy

**Corresponding author:** Giorgia Ghiara, [giorgia.ghiara@gmail.com](mailto:giorgia.ghiara@gmail.com)

**Figure S1.** Score plot of the ED-XRF measurements.


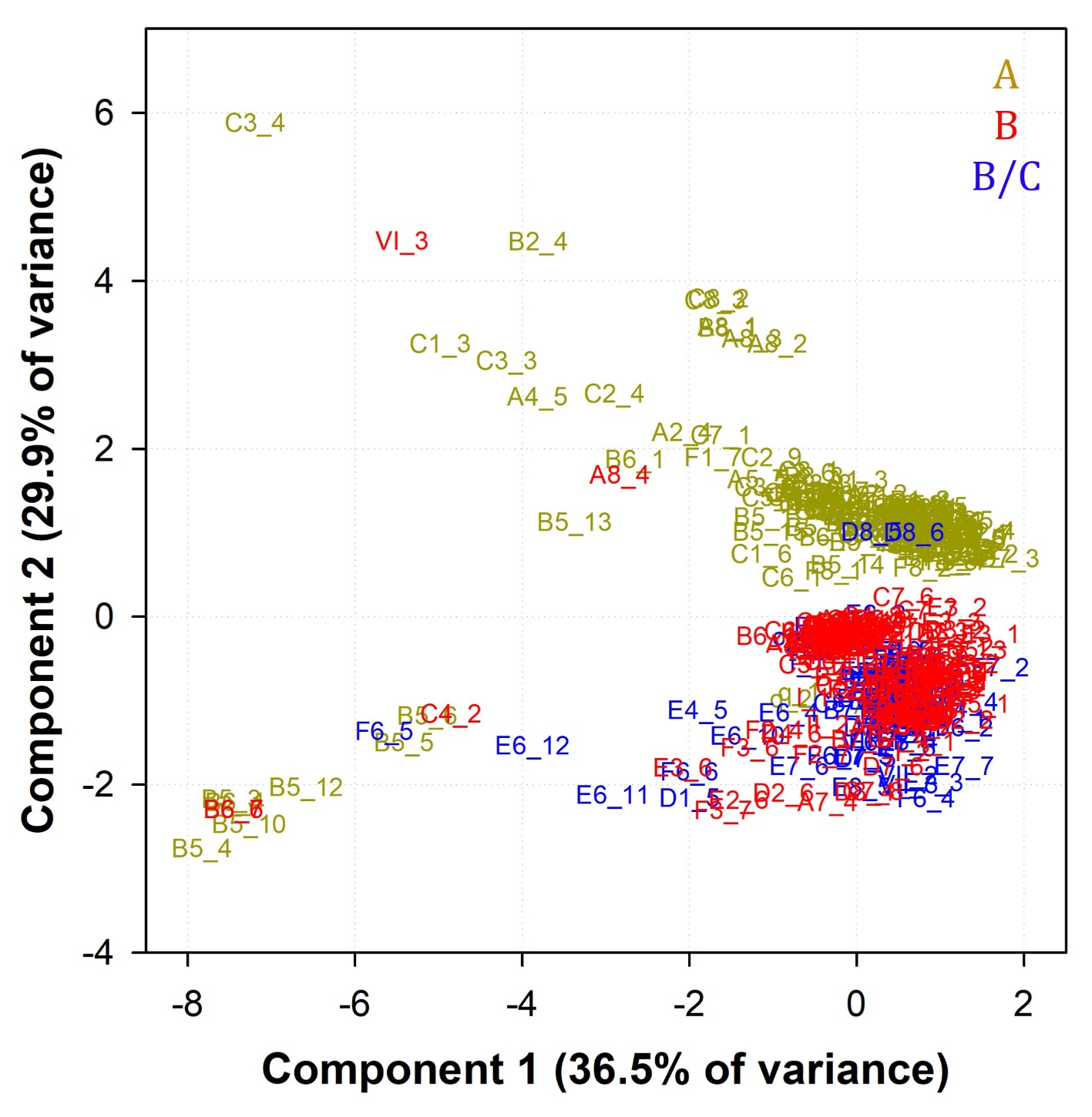

Supplement: Supplementary file 1 — Additional file 1: Figure S1. Biplot of the ED-XRF measurements [file 40494_2024_1143_MOESM1_ESM.docx]
